# Microtiming in Swing and Funk affects the body movement behavior of music expert listeners

**DOI:** 10.3389/fpsyg.2015.01232

**Published:** 2015-08-20

**Authors:** Lorenz Kilchenmann, Olivier Senn

**Affiliations:** School of Music, Lucerne University of Applied Sciences and ArtsLucerne, Switzerland

**Keywords:** microtiming, groove, entrainment, body movement, participatory discrepancies, funk, swing, musical expertise

## Abstract

The theory of Participatory Discrepancies (or PDs) claims that minute temporal asynchronies (microtiming) in music performance are crucial for prompting bodily entrainment in listeners, which is a fundamental effect of the “groove” experience. Previous research has failed to find evidence to support this theory. The present study tested the influence of varying PD magnitudes on the beat-related body movement behavior of music listeners. 160 participants (79 music experts, 81 non-experts) listened to 12 music clips in either Funk or Swing style. These stimuli were based on two audio recordings (one in each style) of expert drum and bass duo performances. In one series of six clips, the PDs were downscaled from their originally performed magnitude to complete quantization in steps of 20%. In another series of six clips, the PDs were upscaled from their original magnitude to double magnitude in steps of 20%. The intensity of the listeners' beat-related head movement was measured using video-based motion capture technology and Fourier analysis. A mixed-design Four-Factor ANOVA showed that the PD manipulations had a significant effect on the expert listeners' entrainment behavior. The experts moved more when listening to stimuli with PDs that were downscaled by 60% compared to completely quantized stimuli. This finding offers partial support for PD theory: PDs of a certain magnitude do augment entrainment in listeners. But the effect was found to be small to moderately sized, and it affected music expert listeners only.

## Introduction

The concept of musical entrainment denotes the power of music to trigger body movement in humans (Clayton et al., 2005; Doffman, 2008; Burger et al., 2014). The concept has been borrowed from physics, and it is mainly used in a scholarly context. In Western beat-oriented genres, the musicians and listeners themselves refer to the effect of music on body movement and emotion (Pfleiderer, 2010) as its “groove” (with respect to Funk, Soul, post-1960s Jazz, Rock; see Pressing, 2002) or “swing” (with respect to older Jazz; see Pfleiderer, 2006).

The causes of entrainment in music listeners remain largely unexplained. This article studies the claim that minute timing discrepancies in the performance of beat-oriented music (Participatory Discrepancies or PDs) trigger entrainment in listeners.

The PD concept, developed by ethnomusicologist Charles Keil, originally had a broad meaning. It denoted a range of deviations that arise in different dimensions of music performance such as timing, pitch, timbre, or dynamics (Keil, 1987). Keil described these deviations as traces of coordinative processes in music performance: “It is the little discrepancies within a jazz drummer's beat, between bass and drums, between rhythm section and soloists, that create swing” (Keil, 1987, p. 277). To Keil, the groove or swing “is not some essence of all music that we can simply take for granted, but must be figured out each time between players” (Keil, 1995, p. 1). Groove, according to Keil, is not part of the composition, but it is created interactively in performance (see also Senn and Kilchenmann, 2011).

In 1995, Keil took notice of microtiming studies by Prögler (1995) and Alén (1995) that gave evidence for the existence of temporal PDs (Keil, 1995). Prögler's and Alén's studies were early examples of a substantial body of timing analyses that, starting in the 1980s, revealed systematic microtemporal patterns in the performance of music in different beat-oriented genres (Rose, 1989; Alén, 1995; Prögler, 1995; Collier and Collier, 1996, 2002; Repp, 1999; Busse, 2002; Friberg and Sundström, 2002; Doffman, 2005, 2008, 2009; McGuiness, 2005, 2006; Benadon, 2006, 2009; Butterfield, 2006; Pfleiderer, 2006; Senn, 2007, 2011; Honing and Haas, 2008; Polak, 2010; Brandmeyer et al., 2011; Kilchenmann and Senn, 2011; Naveda et al., 2011).

Today, the PD concept is used quite exclusively with reference to temporal phenomena only. In his most recent contribution, Keil defined PDs, rather technically, as “measurable differences or discrepancies in attack points and release points along a time continuum” (Keil, 2010). Thus, he consolidated this reduction on timing. But his main idea about the participatory and procedural nature of PDs was still similar to 1987: “The drummer and bassist are consistently in synchrony with each other, but they are also consistently discrepant, different, slightly out of phase or in and out of phase with each other” (Keil, 2010).

In the present study, PDs are defined along Keil's lines. PDs are understood as measureable timing differences between the onsets of musical events arising in a real performance situation: Two professional musicians, a bassist and a drummer, playing a studio jam session, communicating, and interacting with each other musically. Two short excerpts from these performances were used as the base material for creating the stimuli. In these excerpts, all onset timing aspects of the spontaneous performance (i.e., its temporal PDs) were conserved. The treatment manipulations consisted in scaling the magnitude of the PDs, without changing the relationships between the onsets (see Section Stimuli: PD Manipulation).

Timing discrepancies in music performance have been found to arise as a result of intentional variation (Thompson and Luck, 2012), or of limits in sensori-motor synchronization (Aschersleben, 2002; Madison, 2014). They can also be “obligatory” expected deviations related to musical structure (Repp, 1998), or simply mistakes. As defined and operationalized for the present study, PDs are necessarily a combination of temporal variations from different origins. We do not consider this to be problematic as long as no claims are made that attribute the effects of PDs to specific components of the timing deviations.

The claim that temporal PDs are relevant for triggering the groove effect has been readily adopted as a plausible explanation within musical (Berliner, 1994; Monson, 1996) and scholarly communities (Klingmann, 2010). Only recently, empirical research started investigating, whether microtemporal features of a performance do have an actual effect on the listeners' emotional response and/or entrainment behaviors.

Butterfield (2010) tackled the topic from a cognitive discrimination point of view. He studied whether listeners were capable of distinguishing bass lead from drum lead in bass and drums jazz duo recordings, that is whether they recognized when either the bass sounded earlier than the drums or vice versa. The success rate of the participants was barely above chance level. Butterfield concluded that most listeners could not consistently identify PDs of magnitude 30 ms or less (Butterfield, 2010, p. 162). Since PDs occurring in performance are of a similar magnitude, he further concluded that the results offered “little support for the central claims of PD theory” (Butterfield, 2010, p. 165).

Madison et al. (2011) went beyond the question of PD detection to test the groove potential of 100 commercially available tracks from five musical genres (Greek, Indian, Jazz, Samba, and West African). Nineteen listeners rated the perceived groove or entrainment qualities of the tracks (“evokes the sensation of wanting to move some part of the body”). And the authors correlated these ratings with quantitative descriptors of the stimuli, including beat salience, metrical levels, event density, systematic, and unsystematic microtiming. They found correlations between groove ratings and beat salience, and also between groove ratings and event density. The perceived groove qualities of the music examples did not correlate with either of two microtiming scales. The authors concluded that they “could find no support for the idea that microtiming contributes to groove” (Madison et al., 2011, p. 11).

The above two studies question the potential impact of PD theory on music listener response, since (in one case) PDs were not detected, and in the other they did not contribute to a feeling of entrainment. Recent studies conflicted with these results to a degree, finding that PDs can be detected, but suggesting that listeners do not enjoy them. Frühauf et al. (2013) used an online platform to test the effect of microtemporal displacements on the perceived quality of a rock drum pattern. The displacements they applied amounted to 15 or 25 ms in either direction. The perfectly quantized tracks were rated best. Larger displacements resulted in lower ratings, particularly when the displacement was early. These results suggest (at least for rock music) that listeners prefer a perfectly quantized drum performance to one displaying microtemporal deviations. In support of this hypothesis, Davies et al. (2013) found that tracks of Samba, Funk, and Jazz that contained PDs received lower ratings on groove compared to perfectly quantized versions. From this, the authors generally concluded that “microtiming does not increase groove” (Davies et al., 2013, p. 507). Both studies make a case for quantized timing as creating the strongest feeling of groove. We might address this result as the “exactitude hypothesis.”

The most recent paper on this issue was by Madison and Sioros (2014) who studied both the production (musicians) and reception (listeners) side of the groove phenomenon. They instructed musicians to play unfamiliar melodies (in child songs, jazz, or rock style) with maximum or minimum groove and analyzed the impacts on musical parameters like syncopation, the event density on faster metrical levels, or microtiming. They found that the intention to play with maximum groove did alter some aspects of performance (like syncopation), but it did not influence microtiming in a significant way. Using a Brunswickian lens model, the authors studied whether the musicians' intention to play with maximum groove or not was perceived by listeners, and which of the musical parameters cued the listeners to the musicians' intention. Here also, microtiming did not play a relevant role.

In summary, none of the five previous studies offers any support for PD theory: microtemporal deviations appear to be either irrelevant for the groove experience of listeners (Butterfield, 2010; Madison et al., 2011; Madison and Sioros, 2014), or to have a negative effect on groove ratings (Davies et al., 2013; Frühauf et al., 2013). Since the essential effect of groove is to facilitate listeners' body synchronization with a musical beat, PDs appear only to confound the location of the beat and thus diminish the groove potential of music (Merker, 2014).

These empirical results are counterintuitive when related to a large body of statements made by experts in beat-oriented music, such as the professional jazz musicians interviewed by Berliner (1994), Monson (1996) and Doffman (2008). These musicians discuss timing as an essential quality of jazz rhythm, albeit often in metaphorical terms: they talk about playing “ahead” or “behind the beat” (Monson, 1996, p. 56) and thus creating a “subtle, floating quality (Berliner, 1994, p. 154). They speak about the “elasticity” of rhythm (Doffman, 2008, p. 64): the ride cymbal pattern is said to be “solid”, whereas the other drum events are more “liquid” (Monson, 1996, p. 56). The musicians understand grooving as a process of bodily entrainment: it has to “come from your body” (Berliner, 1994, p. 152). When the interaction between musicians is effective, it feels like “walking arm-in-arm with someone” (Monson, 1996, p. 68). And some musicians think that most of the inspiration for playing music originates from the pleasure of body movement itself (Berliner, 1994, p. 152).

One musician, jazz pianist Fred Hersch, explicitly links timing and entrainment: “There's a certain kind of time that's metronomic, that's correct, but doesn't make you want to dance. It doesn't make you want to move, and it doesn't make you want to play” (Berliner, 1994, p. 245). We can summarize that at least some world-class jazz musicians consider flexible timing to be an essential aspect of their craft and that entrainment depends (to a certain degree) on timing. This insider view appears to conflict with the empirical scholarly results presented above.

The present study aimed at empirically testing the central claim of PD theory under the most favorable conditions. It studied whether and how microtemporal alterations influence the body movement behavior of listeners. First, the experimental stimuli were generated from authentic duo performances by expert musicians, played during a jam session in a recording studio. This ensured that the microtemporal deviations complied with the concept of PDs insofar as they were not introduced arbitrarily by the researchers, but generated in a participatory process of authentic music performance. Second, the stimuli were played in two styles, Swing and Funk, that are normally entirely played by human performers (contrasting with other dance-related styles like Disco or Techno that use sequencers) and that are associated with body movement or groove (see Berliner, 1994, for Swing; and Danielsen, 2006, for Funk). Third, the two instruments used were bass and drums, a combination that has been mentioned in several studies as being essential for the creation of groove in beat-oriented music (Keil, 1966; Berliner, 1994; Monson, 1996; Zbikowski, 2004; Butterfield, 2010). Fourth, the responses of the participants were not estimated with psychometric measures through a questionnaire alone. Instead, the participants' actual, beat-related periodic head movement behavior was measured with motion tracking technology that has previously been used successfully in the study of embodied aspects of music performance (Goebl and Palmer, 2009a, 2013) and perception (Burger et al., 2014). Fifth, every participant listened to music examples in only one style, so the effects of the participants' preference for one musical style over the other would not eclipse the PD effects.

In accordance with PD theory, we hypothesized that the music clips with the original PDs would trigger the most intense beat-related periodic head movement in participants (the most entrainment), compared to music clips with either reduced or augmented PD magnitudes. We further hypothesized that music experts would be influenced by the PD manipulations more strongly than non-experts, because we assumed the music experts to be more sensitive to minute temporal aspects of musical sound due to their training. Finally, we hypothesized that the Funk examples would trigger more intense body movement reactions than the Swing examples, because we presumed that Funk was more likely to be played at dance/body movement related events at the time of the experiment than Swing.

## Materials and methods

### Stimuli: Original recordings

Recordings were prepared at Gabriel Studios (Stalden bei Sarnen) with two expert musicians, drummer Dominik Burkhalter, and bassist Wolfgang Zwiauer, on April 19, 2012. The two musicians are professors for their respective instruments at the Jazz Institute of Lucerne University of Applied Sciences and Arts. They pursue international careers as musicians, and they have been playing together in various groups for several years. The tempi of the examples (150 bpm for Swing, 100 bpm for Funk), and the length of the harmonic sequence (12 bars for Swing and 8 bars for Funk) were predefined by the researchers in order to ensure that the stimuli were the same duration in both styles (20 s). The drummer used an acoustic drumset for both recordings; the bassist used an acoustical bass guitar for the Swing recording and an electric bass guitar for the Funk recording. The musicians jammed during several minutes over the harmonic sequences. They heard a metronome click through headphones as a common beat reference, in addition to a monitor mix presenting the playing of the two instruments. The click track was adjusted according to the musicians' wishes: they heard the click on half notes in swing, and on eighth notes in funk. The musicians stated to be comfortable with this setup. The acoustic situation with mutual/self-auditory feedback (Goebl and Palmer, 2009b) can be considered ideal for temporal synchronization between the musicians. The click was also recorded to a separate audio track. After the recording session, the musicians selected those passages of the recordings that, according to their own opinion, had the best groove.

From the musicians' selection, the experimenters chose one iteration of the harmonic pattern of 20 s duration for each of the styles. In these clips, the drum events were replaced by samples of the *Toontrack Superior Custom* & *Vintage* (version 2.3.1) library with sub-millisecond precision using the *Massey Drum Replacement Tool* (version 3.9). All audio editing was performed with *Avid Pro Tools* (version 10.0.0). The drum samples were chosen to match the original drum events in timbre and loudness. (The reasons why the drum events were replaced by samples will be given below; see Section Stimuli: PD Manipulations). The music clips with the replaced drums were sent to the musicians who approved of their aesthetic quality.

The physical onset time of each bass event was measured with millisecond precision using the *LARA* software^1^ and the method described in Kilchenmann and Senn (2011). The physical onset time of each drum event was defined as the starting time of each drum sample, as located by the *Massey Drum Replacement Tool*.

The main idea of stimuli manipulations (see Section Stimuli: PD Manipulations below) consisted in scaling the deviations of the PDs as actually played by the musicians with respect to a deadpan reference version without PDs. This deadpan reference version, or “metronomic grid,” had to be defined first. The grid was conceived as an abstract pattern of equally spaced timings, in which the onset time of every possible event on every metrical level is defined. In both styles, the click onsets of the metronome track provided the anchor points for the metronomic grid.

In Funk (tempo 100 bpm), the musicians heard a click on every eighth note; so the 16th note subdivisions of the grid needed to be defined. The 16th note timing was chosen to subdivide the 8th notes duration (0.300 s) into two equal halves of 0.150 s duration. In the case of the Swing examples (tempo 150 bpm), the metronome clicks sounded on the half notes (backbeats). So on the grid, each half note on a down beat had a duration of 0.800 s and was defined to coincide exactly with a metronome click. The timing of the grid off-beat quarter notes was calculated arithmetically through binary subdivision; each grid quarter note had a duration of 0.400 s and divided the half note into two equal parts. The eighth note level of the metronomic grid in swing was slightly more complicated to establish. Swing traditionally has shuffled eighth notes with a long on-beat eighth and a shorter off-beat eighth. The relationship between the longer and the shorter eighth note are usually expressed as the so-called swing ratio (Friberg and Sundström, 2002). Using a grid with a binary subdivision of quarters into two equal eighth notes (or a swing ratio of 1:1) would have violated an elementary property of swing rhythm. So, a deadpan version with uniformly shuffled eighth notes was created instead. The swing ratio of grid eighths was defined as the mean swing ratio of the timing, played by the musicians in the recorded swing performance; this ratio amounted to 2.66:1. This ratio is in accordance with the results of Friberg and Sundström (2002 p. 337) for a tempo of 150 bpm. We will refer to the deadpan timing of all possible metric positions as the “metronomic grid.”

**Figure 1:**
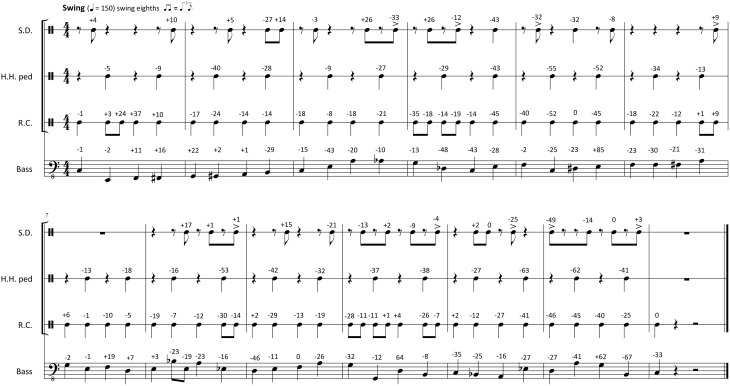
**Transcription of the Swing stimuli with original PDs notated in ms above each note (negative numbers, ahead of metronomic time grid; positive numbers, behind metronomic time grid; S.D., Snare Drum; H.H. ped, foot-operated HiHat Cymbal; R.C., Ride Cymbal)**.

**Figure 2:**
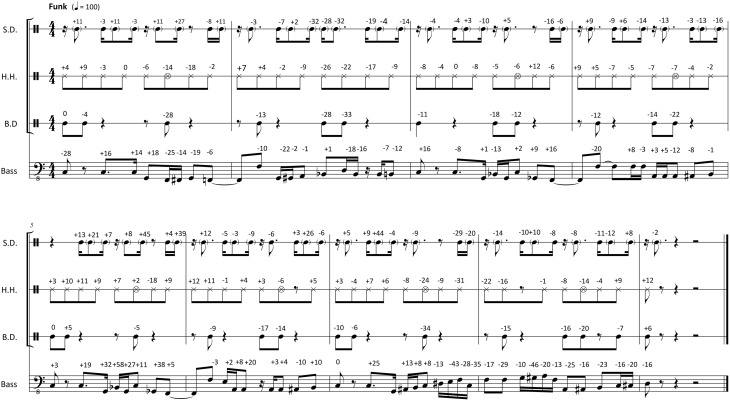
**Transcription of the Funk stimuli with original PDs notated in ms above each note (negative numbers, ahead of metronomic time grid; positive numbers, behind metronomic time grid; S.D., Snare Drum; H.H., hand-operated HiHat Cymbal; B.D., Bass Drum)**.

With the metronomic grid established for both styles, the PD value of each event was defined as the time difference (in milliseconds) between the event's actual physical onset and the timing of the respective position on the metronomic grid. Physical onsets that occurred ahead of the metronomic timing had negative PDs. Late events had positive PDs. Figures 1, 2 show transcriptions of the chosen passages in Swing and Funk style respectively. The PDs of the actual events relative to the metronomic grid are given as milliseconds associated to every note in the transcriptions.

### Stimuli: PD characteristics of original recordings

**Figure 3:**
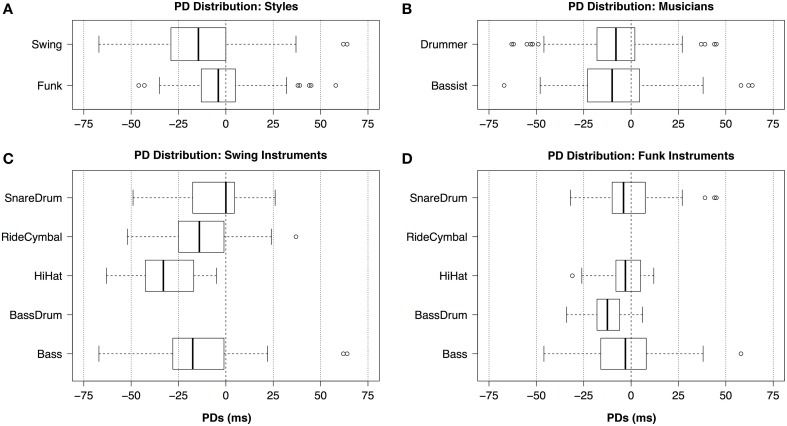
**Boxplots of the original recordings' PD distributions separated by (A) styles, (B) musicians, (C) Swing instruments, (D) Funk instruments**.

The PD characteristics of the selected original recorded passages (as transcribed in Figures 1, 2) are summarized in the boxplots of Figure 3. The boxplots show the differences between the styles, the musicians and the instruments.

There were 157 events in Swing and 214 events in Funk. Since both passages were of the same duration, event density was higher in Funk than in Swing. In the Swing example, the musicians played further ahead of the metronomic grid points (*Median* = −14.5 ms) than in the Funk example (*Median* = −4.0 ms, see Figure 3A). The difference in spread between the PDs in Swing (*SD* = 22.8 ms) and Funk (*SD* = 5.3 ms) implies that the Funk example was generally played “tighter” than the Swing example, which is in accord with the PDs of the stimuli used by Davies et al. (2013). The musicians' choice of different click tracks for the two styles appears to agree with the timing characteristics found in the recordings: for Swing, the musicians requested two clicks per measure, sounding on the offbeats only. For Funk, the musicians requested clicks on every eighth note. So, in Funk, the click track provided the metronomic reference much more frequently than in Swing. This suggests that the Funk click track played a stronger regulatory role on performance timing, compared to the Swing click track.

The two musicians (Figure 3B) were well synchronized with each other in the median. In the selected clips, the bassist (*Median* = −10.0 ms) played slightly ahead of the drummer (*Median* = −8.0). Mood's median test reported this difference as not being significant (χ^2^ = 0.0363, *df* = 1, *p* = 0.547). This observation is relevant for the subsequent analysis: bass onset rise times (the time interval between the physical onset of an event and the moment when it reaches full loudness) are larger than drum event onset rise times. Several theories of rhythm perception link perceptual attack time with the duration of the onset rise time (Vos and Rasch, 1981; Gordon, 1987; Collins, 2006). They suggest that the physical onset of a slowly rising event (like a bass note) must be played early in order to sound in synchrony with a fastly rising event (like a drum stroke). The distribution of PDs indicates, however, that that no such systematic early displacement of the bass events took place in the recordings used for this study. Generally, the bassist appears to have a “looser” timing (*SD* = 23.4 ms) than the drummer (*SD* = 17.7 ms).

The instruments of the drum set and the bass show several distinct style-related PD characteristics. In Swing (Figure 3C), the Bass (*Median* = −17.5 ms) and the Ride Cymbal (*Median* = −14 ms) appear to be synchronized fairly well. This is in accordance with jazz interaction theory, which postulates that Ride Cymbal and Bass together create the timing reference for the jazz band (Hodson, 2007, pp. 29–30). The foot-operated HiHat Cymbal has a tendency to sound earlier (*Median* = −33 ms) than the Ride Cymbal (*Median* = −14 ms). The Snare Drum (*Median* = 0 ms) instead sounds later than the Ride Cymbal. All four instruments appear to have similar PD spread in Swing, this means that their levels of looseness/tightness are comparable (this is also confirmed by the similar *Interquartile Ranges* of the data shown in the boxplots of Figure 3C).

In the Funk example (Figure 3D), the hand-operated HiHat Cymbal (*Median* = −3 ms), the Snare Drum (*Median* = −4 ms), and the Bass (*Median* = −3 ms) are well synchronized. The Bass Drum (*Median* = −12.5 ms) sounds ahead of the three other instruments in the median. In Funk, the four instruments show differences in PD spread. HiHat Cymbal (*SD* = 10.3 ms) and Bass Drum (*SD* = 10.5 ms) are similarly tight. The performance of the Snare Drum (*SD* = 15.9 ms) and the Bass (*SD* = 18.8 ms) appear to be slightly looser. These observations are in accordance with other analytic findings on funk rhythm: Burns and Farris (1981, p. 9) reported that the HiHat Cymbal acts as a timing reference in Funk to which other instruments synchronize. Furthermore, in slow to medium tempi, the right-hand-operated HiHat has been found to have a more stable and tighter timing than instruments of the drumset that are operated by other limbs of the drummer's body (Fujii et al., 2011, p. 500).

### Stimuli: PD manipulations

For each style, 12 stimuli with manipulated timing were created. The general idea was to scale all PDs relative to the metronomic grid. In one series of six stimuli, each individual PD found in the original recording was reduced (scaled down or, in the musicians, parlance: “tightened”); in another series of six stimuli, each PD was expanded or scaled up (“loosened”).

The timing manipulations made it necessary to replace the recorded drum events by drum samples: the loud instruments of the drumset registered in all microphones during recording. Due to this imperfect sound source separation, the timing manipulations on the original audio would have created undesirable sonic artifacts, which could be avoided by the replacements. No replacements were necessary in the bass track: timing displacements of bass events could be performed without noticeable sonic artifacts using *Pro Tools*' time stretching function.

The scaling of the PDs was carried out in five steps of 20%: The series with reduced PDs included six stimuli with the magnitudes of all PDs reduced by 0, 20, 40, 60, 80, and 100%. The 0% simulus had the same PDs as the original recording (see PD magnitudes in Figures 1, 2). In the 100% stimulus, all PDs were reduced by 100%, so all PDs had a magnitude of zero. This was the perfectly quantized or deadpan version with all physical event onsets sounding exactly on the metronomic time grid.

The series with expanded PDs, accordingly, included six stimuli with the magnitude of all PDs expanded by 0, 20, 40, 60, 80, and 100%. The 0% stimulus, again, has the same PD magnitudes as the original recording. In the 100% stimulus, all PD magnitudes were doubled, so this was the stimulus with the “loosest” timing in the two series.

With two *Styles* (Swing and Funk), two *Directions* of PD manipulation (Reduction and Expansion), and six levels of magnitude change (Δ*-Magnitude*: 0, 20, 40, 60, 80, and 100%), the manipulations resulted in 24 stimuli. The music clips were equalized, and a room simulation (*AudioEase Altiverb*, version 7) was added to achieve a balanced, realistic impression. Finally, the clips were mixed down to two stereo channels. The stimuli can be downloaded from the Supplementary Materials section of this paper.

### Participants

The study was carried out with 160 participants in two groups: 79 participants were considered music experts, because they either had completed a professional music education or were enrolled in a Bachelor of Arts / Master of Arts program in Music or Music Pedagogy at the time of the experiment. The 81 participants of the non-expert group did not meet these criteria, even though some of them had considerable experience as music players or listeners. One hundred and fifty eight of the participants were students at either the Lucerne University of Applied Sciences and Arts or at Lucerne University. Two participants were not affiliated with any university. Eighty two of the participants were female, 78 were male. The mean age of the participants was 24 years.

The participants were recruited through e-mails, or the experimenters visited them in class in person and asked them to sign up for the experiment. The participants received a small remuneration (the equivalent of a movie theater ticket) and they could participate in a lottery to win a tablet computer. The participants were informed that they would take part in a listening experiment about musical rhythm, and that they would be filmed during listening. The exact purpose of the experiment was not disclosed to them. The Ethics Committee of the Canton of Lucerne approved of the design and procedure of the experiment.

### Setup

The experiment took place in a quiet office room at the Music Department of Lucerne University of Applied Sciences and Arts. The participant sat at a desk in front of a personal computer screen (*Microsoft Windows*, version 7). The *Neurobs Presentation* (version 16) software presented questionnaires on the screen and allowed for the participant to trigger the stimuli. Sound was played via the *Presonus Firebox* audio interface on *AKG 271 Mk II* studio headphones. A yellow table tennis ball was mounted on top of the frame of the headphones as an optical reference point. Two *Canon XF-105* cameras were placed at angles of −45° and +45° in front of the participant. They were remotely controlled by *Presentation* using an *Applied Logic Engineering 718 LANC* interface. The cameras recorded only during the time when the participant listened to a stimulus.

### Procedure

The participants took the listening test one at a time. In the pretest phase, the participants read an information sheet about the experiment. The participants were informed that the study was about music perception and that they would be filmed during the experiment; the true goals of the study were not revealed to them. The participants did not receive any instructions concerning their body movement behavior, and there was no information given that their body movement would be measured. The headphones were adapted to the participant in size and playback loudness was adjusted. A gap detection test was performed to assess the acoustic time discrimination faculty of the participant. None of the participants failed this test. Three music examples, similar but not identical to the test stimuli, were used for a test run. After the test run, the participant had the possibility to ask questions about the procedure. When the participant expressed to be familiar enough with the procedure to do the test, the experimenter left the room, and the participant performed the listening test on her/his own. The test proper lasted between 13 and 33 min (*M* = 19.9 min; *SD* = 4.0 min). The whole process with instructions, test runs and the collection of participant-related data lasted around 45 min.

Participants were randomly assigned to the Swing or Funk stimuli. Each of the two style groups had exactly 80 participants. Further, a random procedure was employed to determine, whether the participant would first listen to the series of six stimuli with the reduced PDs, or to the series with the expanded PDs. For each series, the sequence of six stimuli was randomized. After listening to a stimulus, the participant rated the examples with two questionnaires. One measurement tool was a 20 item/3 scales Groove questionnaire that has been developed and validated by project partners at Giessen University. The other questionnaire was the SAM Self-Assessment Manikin with 3 items. Since this paper focuses on the analysis of the video data only, the questionnaire data will not be further analyzed here. For the purpose of this paper, the questionnaires gave the participants a task to fulfill, captured their attention, and distracted them from the technical setup. Between series, the participants filled out the PANAS test, and after the experiment, they filled out the NEO-ffi test. This psychometric data will also be analyzed in a future article.

### Measures

The dependent variable of the experiment was the beat-related periodic movement intensity of the optical mark on the headphones, which moved whenever the participant's head moved. The head movement was used as a measure of bodily entrainment, since head bobbing is a common reaction to beat-oriented music. It was preferred to other options (like foot or finger tapping) for several reasons: the head movement was easy to measure with an equipment of only two cameras, the participant's head was free to move at any time during the experiment, and the rest of the equipment (computer screen, table) would not get in the way between the cameras and the participant's head.

The movement of the optical mark was measured as follows: The *Mikromak WinAnalyze* (version 2.5.0) motion tracking software package was used to interpret the two synchronized video signals. Each 40 ms, it calculated the mark's position in space to a precision of 2 mm. The absolute velocity of the mark at any given time was calculated from the mark's trajectory. This resulted in a time series of absolute velocities during the duration of each music example.

The first and last 2 s of these time series were removed from the signal. A short fade-in and fade-out was applied using a sigmoid function. The removal of this part of the signal was due to the observed non-music-related movement behavior of many participants (like leaning back in the chair after triggering the playback or grasping the mouse at the end).

A Fast Fourier Transform was performed on the time/velocity data of each listening event, using the *MIT FFTW* (version 3.3) Software; and a frequency spectrum was calculated. Since it was not clear at the beginning of the experiment whether the participants would rather synchronize their head movements to the quarter note or to the half note frequencies of the stimuli, intensities for both frequencies (±10%) were retrieved. The Funk examples had a tempo of 100 bpm, so the frequency of the quarter notes was at 1.67 Hz and the frequency of the half notes was at 0.83 Hz. The Swing examples had a tempo of 150 bpm, so the frequency of the quarter notes was at 2.50 Hz, and the frequency of the half notes was at 1.25 Hz.

Head movement intensities on the quarter note and half note frequencies were computed for each of the 1908 valid cases (160 participants × 12 stimuli = 1920 cases; the data of one male non-expert participant or 12 cases were invalid). The resulting datasets of head movement intensities were strongly right-skew and leptokurtic. The quarter note and half note datasets became approximately normally distributed after a log-transformation.

The quarter note and half note datasets were highly correlated [*r*_(1906)_ = 0.869, *p* < 0.001]. The quarter note frequency data had a slightly higher mean (*M* = 3.21), than the half note frequency data (*M* = 3.15). This difference was significant [*t*_(3735)_ = 2.108, *p* = 0.035], but the magnitude of the difference was small. The half note frequency data, conversely, had a higher overall variance (*s*^2^ = 0.97) than the quarter note frequency data (*s*^2^ = 0.72); this difference of variance was significant [*F*_(1907, 1907)_ = 1.339, *p* < 0.001]. These features of the datasets suggested that the half note frequency data would record the effects of the PD treatment more clearly than the quarter note frequency data.

Since the music examples in the two styles were played in two different tempi, it was to expect that listeners would rather entrain to the half note frequency for the faster Swing stimuli (150 bpm), and to the quarter note frequency for the slower Funk stimuli (100 bpm). This proved not to be the case: the Funk examples triggered a very similar mean Periodic Head Movement Intensity (*M* = 3.16) on the half note frequency as the Swing examples (*M* = 3.14); the difference between the means was not significant [*t*_(1896)_ = 0.413, *p* = 0.679]. The Funk examples had a slightly higher variance (*s*^2^ = 1.02) on the half note frequency compared to the Swing examples (*s*^2^ = 0.91), but this difference was not significant either [*F*_(947, 959)_ = 1.124, *p* = 0.071]. These comparisons suggest that the tempo differences between the styles had no influence on the means and variances within the quarter note and half note frequency data. Due to its higher variance, the half note frequency data was used as a measure for the participants' periodic entrainment with the music.

## Results

A mixed-design Analysis of Variance was performed in *R* (version 3.0.2). Dependent variable was the log-transformed Periodic Head Movement Intensity on the half note frequency. Independent between-subjects variables were *Style* (Funk, Swing) and musical *Expertise* of the participants (experts, non-experts). Independent within-subjects variables were *Direction* of the PD manipulation (expansion, reduction), and Δ*-Magnitude*, the magnitude of the PD scaling (0, 20, 40, 60, 80, 100%). *Direction* and Δ*-Magnitude* variables governed the PD manipulation: *Direction* determined whether the PDs were up-scaled or down-scaled; Δ*-Magnitude* determined by how many percents the PDs were scaled. The significance level of all statistical tests was set to α = 0.05.

The Shapiro-Wilk test for non-normality, performed on the Periodic Head Movement Intensity data on the half note frequency, was significant (*W* = 0.984, *p* < 0.001). The test has previously been reported to be sensitive to small deviations from normality in large datasets (Field and Hole, 2003, p. 160; Thode, 2002). So, additionally descriptive and visual normality estimates were consulted. Skewness (β_1_ = 0.340) and kurtosis (β_2_ = −0.483) of the Periodic Head Movement Intensity data were both on a small magnitude. The visual inspections of a histogram and of a Q-Q plot did not indicate that the distribution departed from normality in any substantial way. Considering these features of the distribution, the high number of observations (1908 valid cases) and the general claim of the Central Limit Theorem for large datasets (Zabell, 2005; Fischer, 2010), we estimated that the distribution's departure from normality was too small to have a relevant impact on the validity of parametric statistical methods. So, the assumption of normality was sustained for the Periodic Head Movement Intensity data distribution. Levene's test was not significant [*F*_(47, 1860)_ = 0.997, *p* = 0.480], hence homoscedasticity was assumed for the Periodic Head Movement Intensity data. Mauchly's test of sphericity was non-significant for Δ*-Magnitude* (*W* = 0.887, *p* = 0.193) and the Δ*-Magnitude* × *Direction* interaction (*W* = 0.962, *p* = 0.968). Hence, the assumption of sphericity was sustained, and no significance value corrections were applied.

Table 1 shows the complete results of the omnibus mixed Four-Factor 2 × 2 × (2 × 6) ANOVA model; all main effects and interactions have been modeled and are presented in the table. Tables 2–**5** show the results of the *post-hoc* tests. For conciseness, only tests with significant results were reported.

**Table 1:** **Omnibus significance test for all modeled main effects and interactions**.

**Source**	**SS**	**df**	**MSS**	***F***	***p***
**SUBJECT**					
Style	0.2	1	0.166	0.033	0.856
Expertise	0.1	1	0.103	0.021	0.886
Style × Expertise	3.9	1	3.945	0.792	0.375
Error	772.2	155	4.982		
**SUBJECT × DIRECTION**					
Direction	2.4	1	2.418	3.189	0.076
Direction × Style	0.0	1	0.005	0.007	0.935
Direction × Expertise	0.9	1	0.910	1.201	0.275
Direction × Style × Expertise	1.9	1	1.866	2.462	0.119
Error	117.5	155	0.758		
**SUBJECT ×Δ-MAGNITUDE**					
Δ-Magnitude	1.4	5	0.277	0.509	0.770
Δ-Magnitude × Style	3.1	5	0.626	1.151	0.332
Δ-Magnitude × Expertise	0.9	5	0.189	0.374	0.884
Δ-Magnitude × Style × Expertise	4.1	5	0.821	1.508	0.185
Error	421.8	775	0.544		
**SUBJECT × DIRECTION ×Δ-MAGNITUDE**					
Direction × Δ-Magnitude	2.9	5	0.586	0.923	0.465
Direction × Δ-Magnitude × Style	0.9	5	0.186	0.292	0.917
Direction × Δ-Magnitude × Expertise	13.7	5	2.740	4.317	0.001^**^
Direction × Δ-Magnitude × Style × Expertise	4.6	5	0.928	1.462	0.200
Error	491.9	775	0.635		
Total	1844.4	1867			

*Dependent Variable, Log-transformed Head Movement Intensity on half note frequencies. Between-subjects Independent Variables, Style (Swing, Funk); Expertise (Expert, Non-Expert). Within-subject Independent Variables, Direction (Reduction, Expansion); Δ-Magnitude (0, 20, 40, 60, 80, 100%). Subject, repeated measures grouping variable (participants). SS, sum of squares; df, degrees of freedom; MSS, mean sum of squares; F, F-value; p, significance*.

Significance codes: ^***^p < 0.001;

** *p < 0.01*;

^*^p < 0.05.

**Table 2:** *****Post-hoc*** significance test for the expert listeners subgroup**.

**Source**	**SS**	**df**	**MSS**	***F***	***p***
Direction	3.1	1	3.141	5.559	0.019^*^
Δ-Magnitude	0.5	5	0.095	0.169	0.097
Direction × Δ-Magnitude	13.3	5	2.660	4.706	<0.001^***^
Subject	388.0	78	4.974		
Error	484.9	858	0.565		
Total	889.8	947			

*Abbreviations see Table 1*.

As can be seen from Table 1, the *Direction* × Δ*-Magnitude* × *Expertise* interaction of the overall ANOVA was significant (*p* = 0.001). All other effects were non-significant. Particularly, all main and interaction effects involving the *Style* variable were non-significant. The *Direction* main effect was nearly significant (*p* = 0.076), but since a three-factor interaction involving *Direction* was significant, this main effect has not been further investigated.

In order to perform the follow-up tests, the data set was split according to the levels of the variables involved in the significant interaction. In a first step, the set was divided into the two *Expertise* subsets (experts, non-experts), and Two-Factor ANOVAs were run on these subsets with the two within-subject variables *Direction* × Δ*-Magnitude* as independent variables. As can be seen from Table 2, the *Direction* × Δ*-Magnitude* was highly significant (*p* < 0.001) for the expert subset. In the non-expert subset, no significant effects were measured.

**Figure 4:**
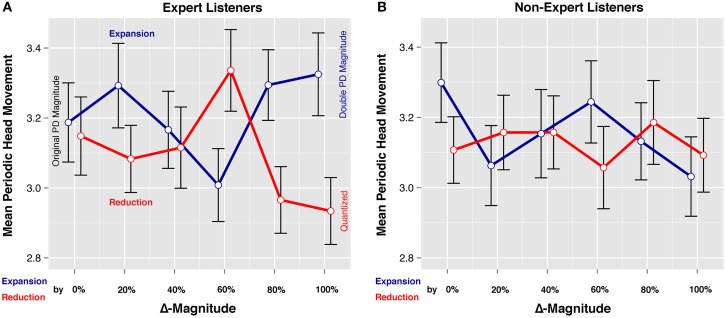
**Interaction plots for (A) music experts and (B) non-experts**. The red graphs represent the mean periodic head movement triggered by clips with reduced PDs (with 0% representing the original PDs and 100% representing the fully quantized timing). The blue graphs accordingly refer to expanded PDs (with 0% representing the original PDs and 100% representing doubled PDs).

The stronger reaction of music experts to the *Direction* and Δ*-Magnitude* manipulations can be confirmed visually in the interaction plots of Figure 4. They show the mean Periodic Head Movement Intensities for the 24 *Direction* × Δ*-Magnitude* × *Expertise* subgroups. The means of the expert listeners are shown in the left plot (A) and the means of the non-experts are shown on the right (B). The red graphs represent the reduction level of the *Direction* variable; hence they stand for the stimuli with diminished PDs. The blue graphs stand for the expansion level of *Direction*, they stand for the stimuli with augmented PDs. The six levels of Δ*-Magnitude* are represented on the horizontal axis. On the leftmost level (0%) the PDs are exactly as played by the musicians in the original recordings. On the rightmost level (100%), PDs have either been reduced completely so that the music is fully quantized (reduction by 100%), or they have been expanded to double magnitude (expansion by 100%).

In a next step, the expert subset was further divided according to *Direction*. Separate follow-up ANOVAs were run on the reduction and expansion subsets. Table 3 shows that there is a significant effect of Δ*-Magnitude* within the reduction subset (*p* = 0.005). In order to localize this effect, a Tukey HSD *post-hoc* test was run to compare the means of all pairs within the six Δ*-Magnitude* levels. The result of the pairwise comparisons can be found in Table 4. The difference between the means of the PD reduction by 60% (*M* = 3.34) and 80% levels (*M* = 2.97) was significant (*p* = 0.012). The effect size (*d* = 0.39) was small to medium according to Cohen's guidelines (Cohen, 1992). The difference between the means of the reduction by 60% (*M* = 3.34) and 100% (*M* = 2.93) levels was also significant and of similar size (*p* = 0.004, *d* = 0.42). We can retain that the music clips with PD magnitudes reduced by 60% triggered significantly higher periodic head movement in experts than the fully quantized clips. No significant effect was measured among the Δ*-Magnitude* levels of the Experts' Expansion subset (*p* = 0.111).

**Table 3:** *****Post-hoc*** significance test for the effect of reduced PDs in expert listeners**.

**Source**	**SS**	**df**	**MSS**	***F***	***p***
Δ-Magnitude	8.2	5	1.645	3.378	0.005^**^
Subject	223.1	78	2.860		
Error	189.9	390	0.487		
Total	421.2	473			

*Abbreviations see Table 1*.

**Table 4:** *****Post-hoc*** pairwise Tukey HSD tests for the effect of reduced PDs in expert listeners**.

**Δ-Magnitude (Mean)**	**20% (3.08)**		**40% (3.12)**		**60% (3.34)**		**80% (2.97)**		**100% (2.93)**	
	***p***	***d***	***p***	***d***	***p***	***d***	***p***	***d***	***p***	***d***
**0% (3.15)**	0.991	0.07	0.999	0.03	0.539	0.18	0.569	0.20	0.385	0.23
**20% (3.08)**			0.999	0.03	0.205	0.27	0.898	0.14	0.762	0.17
**40% (3.12)**					0.351	0.21	0.758	0.16	0.578	0.19
**60% (3.34)**							0.012^*^	0.39	0.004^**^	0.42
**80% (2.97)**									0.999	0.04

Group means of Periodic Head Movement Intensity are given in parentheses. p, p-value; d, Cohen's d. Significance codes: ^***^p < 0.001;

** *p < 0.01*;

* *p < 0.05*.

Finally, the expert subset was divided according to the six levels of Δ*-Magnitude*; and paired *t*-tests were run to compare each *Direction* level (Table 5). A significant effect was measured on the 60% Δ*-Magnitude* level between the means of the two *Direction* levels: the 60% reduction had a higher mean (*M* = 3.34) than the 60% expansion (*M* = 3.01); this difference was significant (*p* = 0.011) and small to medium in size (*d* = 0.33). The situation was reversed on the 80% Δ*-Magnitude* level: the 80% expansion had a higher mean (*M* = 3.29) than the 80% reduction (*M* = 2.97). This difference was significant (*p* = 0.004) and small to medium sized (*d* = 0.38). This change in situation can be assessed visually in Figure 4A: in the interaction plot, the red (reduction) and the blue (expansion) *Direction* graphs cross steeply between the 60% and 80% Δ*-Magnitude* levels. A similar effect was measured on the 100% Δ*-Magnitude* level: the 100% expansion has a higher mean (*M* = 3.32) than the 100% reduction (*M* = 2.93). This effect was significant and its size was small to medium (*p* = 0.001, *d* = 0.41). So, the perfectly quantized stimuli triggered less periodic head movement in music experts than the stimuli with the loosest timing. As can be seen from Table 5, all *t*-tests performed on the other subgroups of Δ*-Magnitude* were non-significant.

**Table 5:** *****Post-hoc*** paired ***t***-tests for expert listeners**.

**Δ-Magnitude (%)**	**Direction**		***t***	**df**	***p***	***d***
	**Reduction**	**Expansion**				
0	(3.15)	(3.19)	0.301	78	0.765	0.04
20	(3.08)	(3.29)	1.645	78	0.104	0.22
40	(3.12)	(3.17)	0.386	78	0.701	0.05
60	(3.34)	(3.01)	−2.616	78	0.011^*^	0.33
80	(2.97)	(3.29)	2.992	78	0.004^**^	0.38
100	(2.93)	(3.32)	3.363	78	0.001^**^	0.41

Group means of Periodic Head Movement Intensity are given in parentheses. t, t-statistic; df, degrees of freedom; p, p-value. Significance codes: ^***^p < 0.001;

** *p < 0.01*;

* *p < 0.05*.

The results were controlled for sex and age of the participants. Sex had a significant (*p* = 0.008) but small (*d* = 0.12) effect on the participants' head movement behavior: the male participants showed a slightly stronger mean response (3.20) than the female participants (3.08). Age was positively correlated with the head movements, but this correlation was weak (*r* = 0.11, *p* < 0.001).

We can summarize that the PD manipulations (*Direction*, Δ*-Magnitude*) only had an effect on the music experts, but not on the non-experts. Effects were only measured on the levels with large Δ*-Magnitude* values (60% and higher), and effect sizes were all small to medium.

## Discussion

In this study, we asked whether the upscaling or downscaling of Participatory Discrepancies (PDs) in Swing or Funk music examples had a significant and measureable effect on listeners' bodily entrainment. We hypothesized that the music clips with the original PDs (as performed by the musicians) would trigger the strongest entrainment, compared to music clips with altered PDs. We further hypothesized that music experts would react more strongly to the manipulations than non-experts and that the Funk stimuli would trigger stronger entrainment than the Swing stimuli.

The second hypothesis is the only one that is supported by the data: it offers evidence that the music experts reacted more strongly to the PD manipulations than the non-experts. The different reactions of the *Expertise* groups presumably depend on differences in training and experience: the music experts have many years of musical training, and they face timing synchronization tasks on a daily basis. We expect that, in the average, the conscious or subconscious faculties for perceiving and discriminating minute timing aspects in music are more developed in music experts than in the general public.

The dependence of the effects on musical *Expertise* challenges PD theory as a universal framework for explaining the musical entrainment phenomenon in general. Many human beings (concert-goers, dance club visitors, sportspeople, workers) engage in rhythmic activities every day and feel the effects of musical entrainment, regardless of their musical expertise. If PDs are irrelevant for the entrainment of music non-experts, other musical (or extra-musical) factors must be relevant instead—candidate variables will be discussed below.

The findings on the relevance of expertise shed new light on previous results: Butterfield (2010) and Madison et al. (2011) reported that microtiming variations did not have a measureable impact on the participants of their listening experiments. The participants of both studies were non-experts, which may (at least partly) explain why no PD effects were measured. Differences in the experimental setup, however, might have had a strong impact as well: in Madison et al. (2011), for example, the stimuli differed in many, non-controllable ways (genre, tempo, sonority, rhythm, harmony, etc.). In this busy context, minute temporal features might go unnoticed by all kinds of listeners, experts and non-experts alike. Conversely, realistic listening situations tend to be busy; and it is plausible that the effects on the experts found in the present study become weaker or disappear, when the music is taken outside of the laboratory and into the noisy surroundings of everyday music listening.

We found no evidence to support our first hypothesis that the music clips with the original PDs triggered more entrainment than the clips with manipulated PDs. None of the clips with Δ*-Magnitude* at 0% was associated with high intensity of periodic head movement. However, the clips with PDs reduced by 60% triggered high entrainment in expert listeners compared to the clips with more strongly quantized timing.

The 60% reduction of PD magnitudes creates music that has a very tight timing: in the original performance, PDs ranged between −67 ms and +85 ms in Swing (*SD* = 22.8 ms), and between −46 ms and +58 ms in Funk (*SD* = 15.3 ms). On the 60% reduction level, these ranges are condensed to −40∕+51 ms (*SD* = 9.1 ms) in Swing and −28∕+35 ms in Funk (*SD* = 6.1 ms). We hypothesize that the expert listeners perceived this kind of tight timing as a result of most competent performance, played by musicians with quasi super-human motor control, coordination, and mutual agreement. On the 80% reduction level, the ranges of the PDs are further reduced to −13∕+17 ms (*SD* = 4.6 ms) in Swing and −9∕+12 ms (*SD* = 3.1 ms) in Funk. On the completely quantized 100% reduction level, the ranges collapse to 0 ms. For the clips on these two levels, entrainment of the expert listeners dropped significantly. Possibly, the highly quantized clips were perceived by the experts as mechanical, machine-like, or not human in comparison with the clip of the 60% reduction level. Such an assumption, however, cannot be substantiated on the basis of the collected data.

Nevertheless, the data indicate that entrainment in expert listeners is significantly weaker in quantized or near-quantised stimuli, compared to the tight stimuli on the 60% reduction level. This result contrasts with the findings of Frühauf et al. (2013) and Davies et al. (2013) who reported the highest groove ratings for the perfectly quantized stimuli compared to stimuli with larger PDs. The “exactitude hypothesis” is not supported by the present research.

The differences of the results of Frühauf et al. (2013), Davies et al. (2013) and the present study probably had several reasons. For one, the measurement methods for groove/entrainment differed quite substantially: In both previous studies the groove measurements were collected through the ratings in a questionnaire. These ratings were based on conscious decisions by the listeners. In the present study, body reactions of the listeners were measured instead. These reactions can, at least to a certain degree, be thought of as unvoluntary and unconscious. As far as we know from research on body language, motor reactions can sometimes convey information that is not communicated through conscious linguistic communication channels; this information may even be eclipsed from the consciousness of the person him- or herself (Birdwhistell, 2010; Porter and ten Brinke, 2010). The analysis of questionnaire data that was also collected during the present study, similar to the data of Frühauf et al. (2013) and Davies et al. (2013), will show whether the discrepancy between conscious vs. unvoluntary reactions persists.

Further, the concepts of PDs differed strongly in the three studies: in the present study, the PDs were the result of actual music performances; and the systematic variations were introduced through scaling of these “natural” PDs. In Frühauf et al. (2013), the microtemporal deviations were artificially introduced by the researchers into an otherwise perfectly quantized environment. Our guess is that the participants perceived these artificial deviations rather as a disturbance of the regular rhythm than as PDs arising in a participatory performance situation. A similar argument applies to the study of Davies et al. (2013): their approach was more sophisticated insofar, as the artificially introduced deviations were (at least partly) derived from timing analyses of real music. But these deviations were applied to the music as fixed patterns that repeated every bar. Consequently the timing of the music clips with greater deviations may have been perceived by the listeners as wobbling rather than as PDs arising in performance. In summary, we suspect that the quantized timing in Frühauf et al. (2013) and Davies et al. (2013) received high groove ratings by the listeners, because the deviation/PD patterns were perceived as disturbances or irregularities.

The present experiment did not offer any evidence to support the hypothesis that the Funk stimuli trigger more beat-related periodic body movement in listeners than the Swing stimuli. The *Style* variable did not account for any significant differences in the participants' body movement behavior.

This said, it would be premature to jump to conclusions about the relative entrainment effects of the two musical styles, based on this experiment alone. The *Style* variable was a between-subjects factor. None of the participants was tested on both genres, and no direct comparisons took place. This was a deliberate design choice by the experimenters and had two reasons: firstly, the experiment would have lasted too long per participant, had they had to rate all 24 stimuli. Secondly, the music examples in Swing and Funk differed in many non-controlled parameters. In direct comparison, these differences could have triggered strong effects in participants that might have eclipsed the effects of the minute systematic PD manipulations of the *Direction* and Δ*-Magnitude* variables. So, all we can conclude from the non-significant results concerning the *Style* variable is that the participants locked into a similar body movement intensity for both styles, regardless of style differences.

The significant differences between the two levels of the *Direction* variable on the 60, 80, and 100% Δ*-Magnitude* levels are difficult to interpret. The simplest case is the 60% level: the clips with PDs reduced by 60% (*SD* = 9.1 ms in Swing and *SD* = 6.1 ms in Funk) triggered more entrainment in expert listeners than the clips with PDs expanded by 60% (*SD* = 36.5 ms in Swing and *SD* = 24.5 ms in Funk). This is in accordance with the notion that tight timing is generally more appreciated by musicians compared to loose or imprecise timing. Hence, the higher entrainment of the experts to the tight stimuli is not surprising.

On the 80 and 100% Δ*-Magnitude* levels, however, the situation is reversed: the expanded PDs triggered significantly more entrainment in experts than the reduced PDs. On the 100% expansion level, the PDs have a large spread (*SD* = 45.6 ms in Swing and *SD* = 30.6 ms in Funk), larger than any displacements used in previous studies. Why did the experts move so strongly to the music with the loosest timing of the whole series? A possible explanation was brought forward by a bassist when he was confronted with the results of the study. He hypothesized that the experts' head movements could be their way of coping with an unclear or fuzzy timing situation in the music. The listeners' coping strategy might consist in projecting their sense of the beat onto an entrained body movement. The coupling between the regular head movement and the inner representation of the beat might strengthen the participants' own sense of the beat, and counteract the unclear signals coming from the music. We might address this head movement as “compensatory”: the head movement is not triggered by a feeling of groove but by the need to stabilize a rhythmically shaky situation. If this tentative explanation is adequate, this would imply that there can be different reasons for listeners to entrain their bodies with music—and that the groove phenomenon might be only one of them.

## Concluding remarks

The present study strikes a new methodological path in groove studies by using authentic PDs generated in actual music performance and by directly measuring the listeners' body movement response (instead of collecting questionnaire data only). Several aspects of the design can be improved: for example, the participants were seated and thus relatively restricted in their body movements. A future study might leave the participants to stand; and the combined movements of feet, legs, torso, and head might accumulate to a greater overall movement (in analogy to Burger et al., 2014). This change might also augment contrasts between groups. Further, musical expertise was defined rather crudely in this study: a participant was considered an expert if she or he was enrolled in or had completed a professional music performance or music pedagogy training. This rough estimation of musical expertise could be replaced by a more accurate test of musicality, e.g., the Goldsmith Music Sophistication Index (Müllensiefen et al., 2014) or the Swedish Musical Discrimination Test (Ullén et al., 2014).

The present study offers some support for PD theory: microtemporal discrepancies arising in music performance do have a measurable impact on the body movement behavior of listeners. But this effect seems to be restricted to music experts only. No sufficient evidence for an effect on the non-experts was found. Also, the effects on the expert listeners were rather small. In the light of these results, the claim that PDs are “most certainly where the power of music comes from: the power to make us listen, make us dance, make us want to participate” (Keil, 1995, p. 9) appears to be hyperbolic. The appreciation of microtiming seems to be an exclusive pleasure for the music elite. For the great majority of listeners, other aspects of music, different from microtemporal PDs, must be relevant for triggering body movement. One important question for the future of groove studies is to identify and test these other aspects. Madison et al. (2011) have proposed several candidate variables; one of them—beat salience—appears to be very likely to correlate positively with body movement intensity: it is a plausible assumption that listeners will best entrain their body movement to a beat they can easily detect. Two recent studies (Sioros et al., 2014; Witek et al., 2014) have found evidence that syncopation is relevant for the perception of groove. Another possible important variable might be the loudness of the music: it is barely a coincidence that loud music is played in places where people move to music.

## Author contributions

Conceived and designed the experiment: LK, OS. Performed the experiment: LK. Analyzed the data: OS. Wrote the paper: OS, LK.

### Conflict of interest statement

The authors declare that the research was conducted in the absence of any commercial or financial relationships that could be construed as a potential conflict of interest.

## Footnotes

^1^Lucerne Audio Recording Analyzer LARA [Computer Software] (2015). Version 2.5.5. Available online at: www.hslu.ch/lara (retrieved 11.05.2015).
